# Mean Platelet Volume/Platelet Count Ratio and Risk of Progression in Glioblastoma

**DOI:** 10.3389/fonc.2021.695316

**Published:** 2021-06-08

**Authors:** Johannes Wach, Stefanos Apallas, Matthias Schneider, Johannes Weller, Patrick Schuss, Hartmut Vatter, Ulrich Herrlinger, Erdem Güresir

**Affiliations:** ^1^ Department of Neurosurgery, University Hospital Bonn, Bonn, Germany; ^2^ Division of Clinical Neurooncology, Department of Neurology and Centre of Integrated Oncology, University Hospital Bonn, Bonn, Germany

**Keywords:** glioblastoma, mean platelet volume, platelet count, progression, ratio

## Abstract

**Objective:**

The mean platelet volume/platelet count (MPV/PC) ratio is an emerging biomarker in selected types of cancer. The objective of this study is to analyze the association of MPV/PC ratio with progression and survival in glioblastoma (GB) patients, with consideration of patient demographics, tumor morphology, extent of resection, molecular pathology, and oncological therapy.

**Methods:**

One hundred ninety-one patients with newly diagnosed GB were analyzed retrospectively. MPV/PC ratio groups (≤ or >0.0575) were dichotomized into low-MPV/PC ratio (≤0.0575) and high-MPV/PC ratio (>0.0575) groups according to the most significant split in the log-rank test.

**Results:**

A two-sided Fisher’s exact test showed no significant differences in the confounders between the low- and high-MPV/PC ratio groups. The median progression-free survival (PFS) was 9.0 months (95% CI=8.0–10.0) in the low-MPV/PC ratio group (n=164) and 6.0 months (95% CI=3.0–8.9) in the high-MPV/PC group (n=28) (*p*=0.013). Multivariate Cox regression analysis including the O-6-methylguanine-DNA methyltransferase (MGMT) status, age (≤/>65 years), baseline Karnofsky Performance Status (KPS), and MPV/PC ratio showed high-MPV/PC ratio as a predictor of progression (*p* =0.04, HR=1.61, 95% CI=1.01–2.57). In the subgroup of IDH1 wild-type GBs, high MPV/PC ratio was still a significant predictor for shortened PFS (*p*=0.042, HR=1.60, 95% CI=1.02–2.52). MPV/PC ratio showed no significant effect in the overall survival (OS) analysis. Median OS was 15.0 months in the high-MPV/PC ratio group and 21.0 months in the low-MPV/PC ratio group (*p*=0.22).

**Conclusion:**

MPV/PC ratio may independently predict the progression-free survival rates of patients with glioblastoma multiforme.

**Graphical Abstract:**
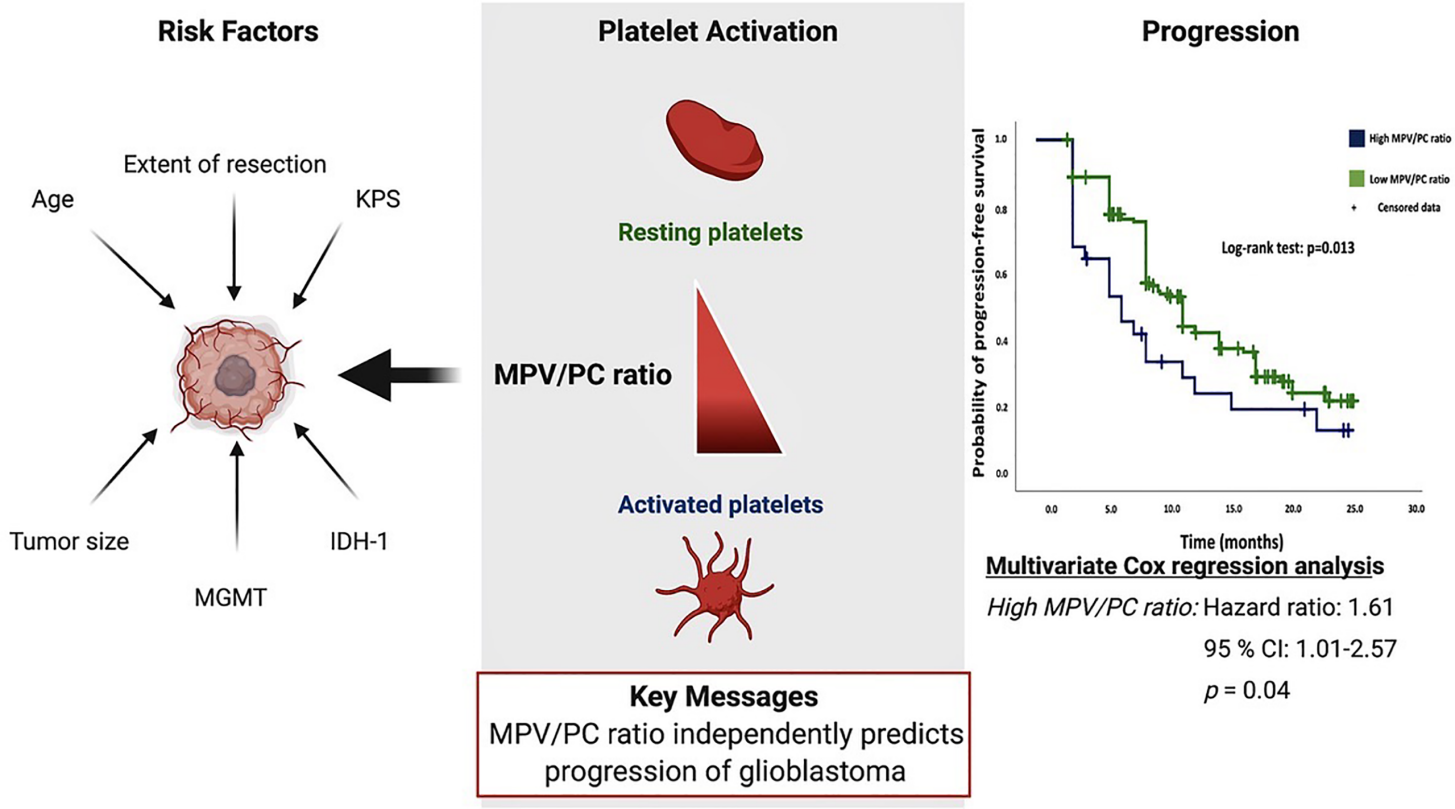


## Introduction

Glioblastoma (GB) remains a fatal disease with low survival rates, accounting for 15.8% of all brain and central nervous system (CNS) tumors (1). A variety of predictors exist for long-term survival in GB, including female sex, young age, and Karnofsky Performance Status (KPS) of 80 or higher at the time of diagnosis (2–4), as well as mutations in several biological markers, e.g., hypermethylation of the O-6-methylguanine-DNA methyltransferase (*MGMT*) promoter, mutations in isocitrate dehydrogenase (*IDH*)-1 codon 132, and mutations of the promoter *TERT* gene (5–8).

Standard treatment includes microsurgical resection with functional preservation followed by concomitant radiochemotherapy, with a strong positive correlation between the extent of resection (EOR) and prolonged overall survival/progression-free survival (PFS) (9, 10). The prognostic benefits and importance of a concomitant radiochemotherapy regimen including temozolomide (TMZ) or lomustine-TMZ are evident (6, 11).

The potential role of platelet–immune cell interaction and its specific role in glioma resistance and tumor progression were recently debated. The known interaction between immune cells and platelets necessitates the elucidation of new and potential platelet biomarkers for upcoming and promising immunotherapeutic approaches (12).

Mean platelet volume (MPV) reflects the size and activity of platelets and is related to different kinds of tumor (13–15). In addition, a strong inverse relationship exists between MPV and platelet count (PC). Mean platelet volume/platelet count ratio is a feasible marker for the activity of platelets and was suggested to be a useful predictor of survival in esophageal squamous cell carcinoma and pancreatic cancer (16, 17). High MPV and low PC levels were found to be correlated with vascular invasion in colorectal cancer patients (18). The role of the MPV/PC ratio in survival and progression of glioblastoma has not been investigated so far. Therefore, the objective of this study was to analyze the association of the baseline MPV/PC ratio prior to initial surgical resection with the known predictors on survival: PFS and KPS, in patients with newly diagnosed GBs treated with maximum safe resection and subsequent molecular-biologically-driven diagnostics and treatment from 2013 to 2018.

## Methods

### Study Design and Patient Characteristics

This study follows the guidelines set forth by the Strengthening the Reporting of Observational Studies in Epidemiology (STROBE) statement. Ethical approval was received by the ethics committee of the University of Bonn. Patient data were pseudonymized before assessment and publication. Informed consent was not necessary and waived by the local ethical committee of the University of Bonn as a retrospective design was used.

From May 2013 to December 2018, 348 patients with GB were treated surgically and were analyzed retrospectively. Inclusion criteria for the present study were histopathologically confirmed GB, age greater than 18 years, availability of survival information and KPS, single intracranial contrast-enhancing tumor lesion, patients treated by neurosurgical resections *via* craniotomy with an extent of resection ≥90%, and completion of postoperative radiotherapy and concomitant temozolomide chemotherapy or first course of CCNU/temozolomide (11). Excluded from further analyses were 156 patients who underwent biopsy only, without additional cytoreductive surgery, patients with multiple intracranial lesions, partial or no postoperative oncological therapy, and patients with no clinical follow-up (≥1 month). Decision making process to perform a biopsy were made at the initial presentation and were based on our previously described indications (19).

### Surgical Procedure

Surgical resection was performed using a white-light resection under neuronavigation guidance (Brainlab Curve, BrainLAB AG, Feldkirchen, Germany) and 5-aminolevulinic acid (5-ALA 20 mg/kg, Gliolan; Medac GmbH, Wedel, Germany)-guided surgery, as described previously (19). Postoperative MRI was performed within 72 h after surgery by a senior neuroradiologist to investigate the extent of resection. Gross total resection is defined as a resection without residual Gd-enhancement, whereas subtotal resection is any resection with residual Gd-enhancement and an extent of resection ≥90%.

### Immunohistochemistry

Histological evaluation was conducted according to the World Health Organization 2016 diagnostic consensus criteria (19, 20). Paraffin sections were stained with hematoxylin and eosin (H&E). Sections were examined immunohistochemically with Molecular Immunology Borstel-I (MIB-I) antibody, glial fibrillary acidic protein (GFAP), and IDH1 (19). MGMT status was determined by methylation-specific polymerase chain reaction (PCR) and reported according to Hegi et al. (5).

### Biochemical Measurements

Retrospective data were acquired using the laboratory information system Lauris (version 17.06.21, Swisslab GmbH, Berlin, Germany). Venous blood samples were routinely collected within 24 h prior to onco-surgical resection of GB. These laboratory investigations were performed at constant time intervals, which enabled the analysis of patients’ overall survival and progression-free survival rates. The routine examination before surgery included complete blood count, and kidney and liver tests. A coagulation profile (INR, aPTT) was also examined in every patient. Platelets were evaluated by the determination of MPV and platelet count. The MPV/PC ratio was calculated as the MPV measured in 10^−15^ L divided by the absolute platelet count measured in ×10^9^/L. MPV/PC ratio calculation was based on the laboratory values (MPV&PC) determined at admission within 24 prior to initial surgery.

### Tumor Morphology

#### Tumor Characteristics & Peritumoral Edema

Tumor characteristics were evaluated based on the tumor area (21), maximum extent of peritumoral edema (22), and tumor location classified according to the topography using the Sawaya grading system (23). Definitions and measurements of the characteristics pertaining to tumor morphology have been described in our previous study (19).

### Follow-Up & Treatment

Post-surgery treatment protocols were evaluated and determined at the local tumor board review. Follow-up MRI was routinely performed every 3 months. Decision making and definitions of glioblastoma progression were based on the Response Assessment in Neuro-Oncology (RANO) criteria as actualized in 2017 (24). Overall survival (OS) was defined as survival time after the date of primary diagnosis in months.

### Statistics

We used the Fisher’s exact test (two-sided) for analysis of nominal variables and the Student’s *t*-test for metric variables for a comparison of the low- and high-MPV/PC ratio groups. Only two-sided *p*-values were reported in the present study. Kaplan–Meier charts of OS and PFS were created. The optimal cutoff point of MPV/PC ratio for dichotomization of this continuous variable was defined using the minimum *p*-value approach (25). The optimal cut-point is the one that results in a minimum *p*-value of the log-rank test regarding the progression of GB. Differences between the high- and low-MPV/PC ratio groups were analyzed using the log-rank test. A *p*-value < 0.05 was defined as statistically significant. A multivariate Cox regression analysis was performed to analyze the PFS and OS in the entire cohort as well as for IDH1 wild-type glioblastomas. Data were organized and analyzed using SPSS^©^ for MacOS 10.15 version 25.0 (IBM Corp, Armonk, NY, USA).

## Results

### Patient Characteristics

We analyzed the 191 patients that fulfilled the inclusion criteria. Median age (25th–75th percentiles) was 62.0 years (52.5–70.0 years) and the patient population showed a male predominance (female:male = 1:1.66). The mean MPV/PC ratio was 0.0442 (standard deviation (SD): ± 0.0152). MPV/PC ratio groups were dichotomized into low-MPV/PC ratio (≤0.0575) and high-MPV/PC ratio (>0.0575) groups according to the most significant split in the log-rank test (p = 0.013) regarding PFS (**Table 1**). A total of 163 (85.3%) patients had a low-MPV/PC ratio and 28 patients (14.7%) had a high-MPV/PC ratio. Age, sex, body mass index (BMI), preoperative KPS, presence of another extracranial neoplasia dexamethasone intake, tumor area, maximum diameter of peritumoral edema, Molecular immunology borstel-(MIB)I index values, rate of tumors located in the eloquent area, IDH1 mutation, and MGMT promoter hypermethylation status were homogenously distributed between both MPV/PC ratio groups. Due to a potential influence of corticosteroids on inflammatory parameters and MPV/PC ratio, further analysis of metric data using independent t-test was performed. The mean (+/- SD) MPV/PC ratio of patients who received corticosteroids for 7 days or longer was 0.043 +/- 0.017, whereas patients who have underwent corticosteroid therapy for less than 7 days had a mean MPV/PC ratio of 0.046+/- 0.013 (*p* = 0.23). Mean (+/- SD) baseline serum c-reactive protein (CRP) level was 3.17 +/- 8.14 in patients with corticosteroid intake for ≥ 7 days, and patients who received corticosteroids for less than 7 days had a mean (+/- SD) serum CRP level of 2.81 +/- 4.30, respectively (*p* = 0.73).The baseline patient characteristics and analyses by two-sided Fisher’s exact test and independent t-test are summarized in **Table 2**.

**Table 1:** Cutoff points of Mean Platelet Volume/Platelet Count ratio for dichotomization using log-rank test split regarding the progression of glioblastoma.

Cut-Off	Number (Low-/High-MPV/PC Ratio Group)	*p*-Value
≤/>0.0585	170/22	0.029
≤/>0.0580	168/24	0.025
**≤/>0.0575**	**164/28**	**0.013**
≤/>0.0570	163/29	0.12
≤/>0.0565	161/31	0.11
≤/>0.0560	158/34	0.08
≤/>0.0555	155/37	0.06
≤/>0.0550	153/39	0.12
≤/>0.0545	151/41	0.09
≤/>0.0540	150/42	0.14

MPV, Mean Platelet Volume; PC, Platelet Count.

The bolded text shows the optimal cut-off point.

**Table 2:** Comparison of low- versus high-Mean Platelet Volume/Platelet Count ratio group (using Fisher’s exact test (two-sided) and independent *t*-test).

Characteristics	High-MPV/PC Ratio (28/191; 14.7%)	Low-MPV/PC Ratio (163/191; 85.3%)	*p*-Value
Age (years)			
≤65	12 (42.9%)	103 (63.2%)	
>65	16 (57.1%)	60 (36.8%)	0.06
Sex			
Female	6 (21.4%)	66 (40.5%)	
Male	22 (78.6%)	97 (59.5%)	0.06
Body mass index (mean ± SD)	25.96 ± 3.7	28.15 ± 16.00	0.49
Preoperative KPS			
≥70	27 (96.4%)	158 (96.9%)	
<70	1 (3.6%)	5 (3.6%)	0.99
Secondary malignant neoplasm			
Present	2 (7.1%)	14 (8.6%)	
Absent	26 (92.9%)	149 (91.4%)	0.99
Dexamethasone intake			
≥7 days	10 (35.7%)	86 (53.1%)	
<7 days	18 (64.3%)	76 (46.9%)	0.31
Tumor area (mean ± SD), mm^2^	1353.99 ± 1113.8	1399.16 ± 947.1	0.84
Peritumoral edema (mean ± SD), mm	24.8 ± 13.4	22.9 ± 10.4	0.33
Sawaya			
1	3 (10.7%)	23 (14.1%)	
≥2	25 (89.3%)	140 (85.9%)	0.77
Extent of resection			
GTR	19 (67.8%)	127 (77.9%)	
STR	9 (32.1%)	36 (22.1%)	0.24
IDH-1 mutation (available in 181 patients)	1/26 (3.8%)	15/155 (9.7%)	0.48
MGMT promoter hypermethylation (available in 187 patients)	13/28 (46.4%)	84/159 (52.8%)	0.55
MIB-I labeling index (mean ± SD),	18.33 ± 7.76	15.96 ± 7.38	0.15

MPV, Mean Platelet Volume; PC, Platelet Count; SD, Standard deviation; KPS, Karnofsky Performance Status; GTR, Gross total resection; STR, Subtotal resection; IDH-1, Isocitrate dehydrogenase-1; MGMT, O-6-methylguanine-DNA methyltransferase; MIB-1, Molecular Immunology Borstel-1.

### Progression-Free Survival Outcomes in Low- and High-MPV/PC Ratio Groups

The median clinical follow-up time was 15.0 months (25th–75th percentiles, 9.0–22.0 months), with a median magnetic resonance imaging (MRI) surveillance period of 9.0 months (25th–75th percentiles, 6.0–18.0 months). The median PFS in the entire population was 9.0 months (95% CI = 7.96–10.04, *n* = 191). The 6-, 9-, and 12-month PFS rates after diagnosis were 64.6% (124/192), 38.5% (74/192), and 26.0% (50/192), respectively.

Median PFS in the low-MPV/PC ratio group was 9.0 months (95% CI = 8.0–10.0, *n* = 163), compared to 6.0 months (95% CI = 3.0–8.9, *n* = 28) in the high-MPV/PC ratio group.

**Figure 1:**
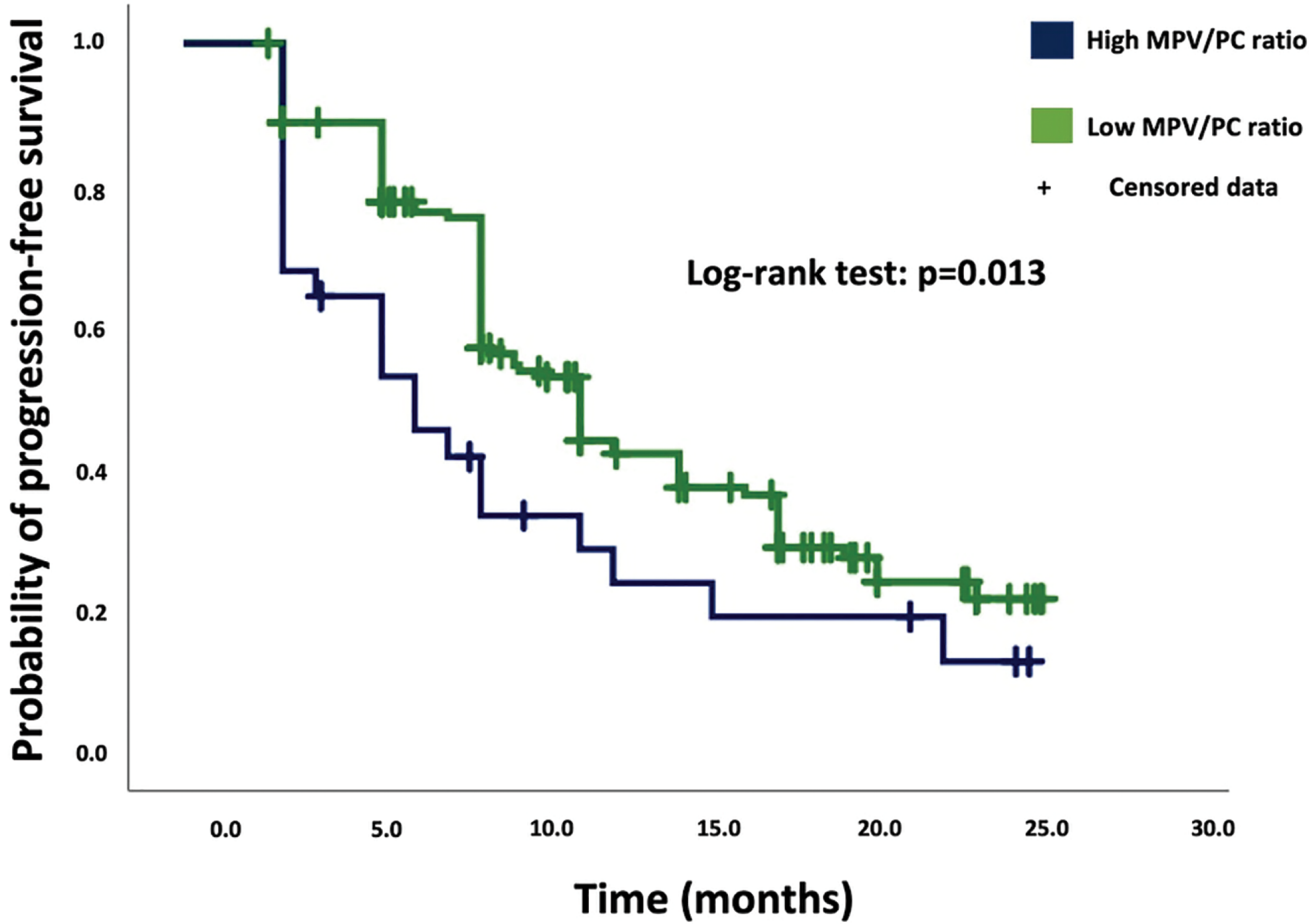
Kaplan–Meier analysis of cumulative progression-free survival stratified by the parameters high-MPV/PC ratio (blue line) and low-MPV/PC ratio (green line). Censored patients (progression-free at last follow-up) are indicated on the curves. The time axis is right-censored at 30 months. *p* = 0.013 (log-rank test).

Based on the Kaplan–Meier method, low-MPV/PC ratio was significantly associated with enhanced PFS compared to high-MPV/PC ratio (log-rank test: *p* = 0.013, **Figure 1**).

Univariate Cox regression revealed a significant association between the high-MPV/PC ratio group and tumor progression [hazard ratio (HR): 1.70, 95% CI = 1.07–2.70, *p* = 0.024]. Univariate analysis showed also a significant association between PFS and MGMT methylation status. The median PFS of GB patients with a hypermethylated MGMT promoter status was 15.0 months (95% CI = 10.66-19.34), and 9.0 months (95% CI = 8.60-9.4) in GB patients without hypermethylated MGMT status (Univariate Cox regression analysis: HR = 2.26, 95% CI = 1.58-3.25, p < 0.001).

Multivariate Cox regression analysis of PFS with consideration of MPV/PC ratio, age (≤65/>65 years), baseline KPS at admission, and MGMT methylation status was performed. The analysis revealed high-MPV/PC ratio as an independent statistically significant predictor for shortened PFS (*p* = 0.044, HR = 1.61, 95% CI = 1.01-2.57) (**Table 3**). Unmethylated MGMT promotor status was also a significant predictor regarding prolonged PFS (*p* < 0.001, HR = 2.26, 95% CI = 1.56-3.28).

**Table 3:** Multivariate Cox regression analysis of progression-free survival.

Entire Cohort			
Variable	Hazard Ratio	95% CI	*p*-Value
High-MPV/PC ratio	1.61	1.01–2.57	0.044
Age > 65 years	1.23	0.84–1.79	0.29
KPS ≤ 70	2.26	0.81–6.29	0.12
Unmethylated MGMT promotor	2.26	1.56–3.28	<0.001
*IDH1 wild-type glioblastoma patients*			
High-MPV/PC ratio	1.60	1.02-2.52	0.042
Age > 65 years	1.29	0.88-1.90	0.19
KPS ≤ 70	1.86	0.66-5.19	0.24
Unmethylated MGMT promotor	2.52	1.71-3.73	<0.001

CI, Confidence interval; MPV, Mean Platelet Volume; PC, Platelet Count; KPS, Karnofsky Performance Status; MGMT, O-6-methylguanine-DNA methyltransferase.

### Progression-Free Survival Outcomes in IDH1 Wild-Type Glioblastoma

One-hundred sixty-five patients had a IDH1 wild-type glioblastoma. The median PFS in IDH1 wild-type GB was 9.0 months (95% CI = 8.26-9.74, *n* = 165). Twenty-five patients had an increased MPV/PC ratio, and 140 patients had a low MPV/PC-ratio. Median PFS in the low-MPV/PC ratio group was 10.0 months (95% CI = 9.06–10.94, *n* = 140), compared to 6.0 months (95% CI = 3.48–8.52, *n* = 25) in the high-MPV/PC ratio group. The Kaplan–Meier method showed that in the subgroup of IDH1 wild-type GB patients low-MPV/PC ratio was also significantly associated with enhanced PFS compared to high-MPV/PC ratio (log-rank test: *p* = 0.014). Univariate Cox regression revealed a significant association between the high-MPV/PC ratio group and tumor progression (HR: 1.67, 95% CI = 1.07–2.61, *p* = 0.025). Univariate analysis revealed an additional significant association between PFS and MGMT methylation status. The median PFS of IDH1 wild-type GB patients with a hypermethylated MGMT promoter status was 15.0 months (95% CI = 11.28-18.72), and 9.0 months (95% CI = 8.62-9.38) (Univariate Cox regression analysis: HR = 2.33, 95% CI = 1.61-3.39, p < 0.001) in IDH1 wild-type GB patients without hypermethylated MGMT status.

Multivariate Cox regression analysis (**Table 3**) of PFS with consideration of MPV/PC ratio, age (≤65/>65 years), baseline KPS at admission, and MGMT methylation status was performed. The analysis revealed high-MPV/PC ratio (*p* = 0.042, HR = 1.60, 95% CI = 1.02-2.52), and MGMT unmethylated promotor status (*p* < 0.001, HR = 2.52, 95% CI = 1.71-3.73) as independent predictor for prolonged PFS in IDH1 wild-type GB.

### Overall Survival in Low- and High-MPV/PC Ratio Groups

The median OS in the entire study population was 20.0 months (95% CI = 17.6–24.4). Patients with GB in the high-MPV/PC ratio group had a median OS of 15.0 months (95% CI = 10.6–19.4), compared to 21.0 months (95% CI = 17.6–24.4) in the low-MPV/PC ratio group (log-rank test, *p* = 0.22). Log-rank tests revealed hypermethylated MGMT promotor status (*n* = 97; median OS (95% CI): 27.0 (22.7–31.3) vs. 18.0 (16.6–19.3), log-rank test: *p* < 0.001) and an age ≤ 65 years (*n* = 121; median OS (95% CI): 22.0 (18.7–25.2) vs. 18.0 (14.2–21.8), log-rank test: *p* = 0.023) as significant predictors for prolonged OS.

Multivariate Cox regression analysis with consideration of MGMT promotor status, age, and KPS at admission showed that hypermethylated MGMT promotor status (*p* < 0.001, HR: 2.42, 95% CI = 1.66–3.51) and an age ≤ 65 years (*p* < 0.001, HR: 2.01, 95% CI = 1.44–3.07) were independent significant predictors regarding prolonged OS. The MPV/PC ratio was not a significant predictor in the multivariate analysis of OS (*p* = 0.74, HR: 1.09, 95% CI = 0.65–1.81).

### Clinical Outcome

**Figure 2:**
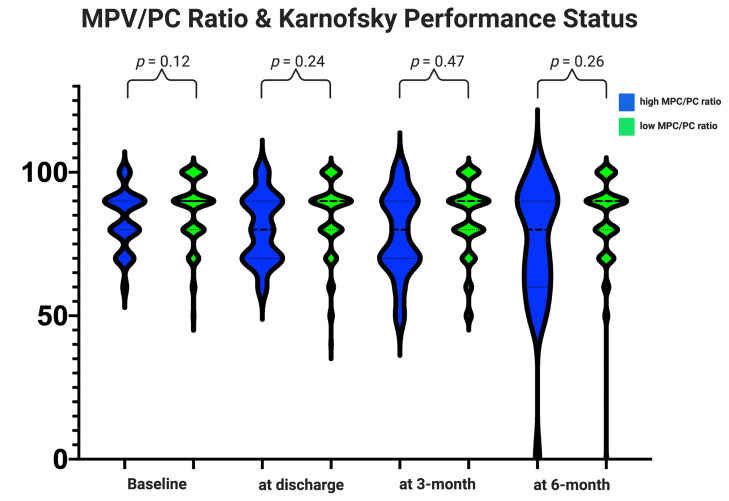
Functionality according to the Karnofsky Performance Status among the MPV/PC ratio groups. Violin plots depicting the degree of functionality according to the Karnofsky Performance Status in the period from admission to the follow-up examination at 6 months stratified by the parameters high-MPV/PC ratio (blue) and low-MPV/PC ratio (green). Violin plots shows mean and distribution of Karnofsky Performance Status. The thick black lines are the median values. (*p*-values of the Student’s *t*-test).

Baseline KPSs were homogenously distributed among both MPV/PC ratio groups (**Table 2** and **Figure 2**). Comparison of the mean values of Karnofsky Performance Status from initial status at admission until the follow-up examination at 6 months using an independent *t*-test did not reveal significant differences (**Figure 2**).

The slightly poorer clinical course of the patients with a high-MPV/PC ratio did not significantly influence the completion of the postoperative treatment protocols. We found that 33 (33/163; 20.2%) patients in the low-MPV/PC ratio group and 10 (8/28; 28.6%) in the high-MPV/PC group had a preterm interruption of the postoperative adjuvant therapy (Fisher’s exact test (two-sided): *p* = 0.32).

## Discussion

We found that a high-MPV/PC ratio correlates with progression of GB and is an independent risk factor for progression-free survival. Our findings suggest the potential importance of assessing the prognosis of GB by combining clinicopathological characteristics with MPV/PC ratio.

Recently, increasing attention has been paid to the role of the MPV, which is known as an early marker of the activation of platelets. Platelets with elevated MPV contain more α-granules and release more prothrombotic factors, which aggravate inflammation (26). Elevated MPV levels are observed in a variety of malignancies, such as esophageal squamous cell carcinoma, pancreatic cancer, hepatocellular carcinoma, gastric cancer, papillary thyroid carcinoma, and colorectal cancer (16, 17, 27–30). In a cohort of 509 colorectal carcinoma patients, Li et al. observed that elevated MPV was an independent marker of poor overall survival (30). However, previous studies reported that MPV is inversely correlated with the platelet count in normal subjects (31). Therefore, a few studies have suggested that MPV/PC ratio is a superior index to MPV alone in some diseases (17, 27, 32).

To date, no study has examined the relationship between MPV/PC ratio and GB survival parameters. Univariate analysis using two-sided Fisher´s exact test showed no heterogeneous distribution regarding demographics, comorbidities, physical condition, histopathology and molecular pathology among the MPV/PC ratio groups. Our data showed that patients with a baseline elevated MPV/PC ratio prior to initial surgery had a significantly shorter PFS of GB in uni- and multivariate analyses. Therefore, baseline MPV/PC ratio seems to be independently correlated with progression-free survival. In contrast to MPV/PC ratio, MPV alone was not a significant predictor for progression-free survival or overall survival in both uni- and multivariate analyses. Alimohammadi et al. (33) performed a retrospective analysis with regard to the role of platelet distribution width (PDW)-to-platelet count ratio in the prediction of overall survival in glioblastoma in 143 cases. They found that an elevated PDW/PC ratio is an independent predictor of shortened overall survival. Therefore, this study also confirmed a relationship between the platelet activity and survival outcome in glioblastoma. However, this study did not analyze progression-free survival and the role of the MPV/PC ratio in the prediction of overall survival or progression-free survival. Furthermore, data regarding molecular pathology (IDH-1 & MGMT) are not available in this study and were not considered for the multivariate analysis. Statistical analysis of overall survival in our study revealed no significant relationship between overall survival and MPV/PC ratio in our series. The shorter PFS despite no statistically significant association between OS and MPV/PC ratio could be explained by the slightly poorer general state that is reflected in the course of the KPS of the high MPV/PC ratio group. Furthermore, those patients with a decreased and non-ambulatory general state who have a progress of GB and without the intention for further treatments often do not pass the follow-up visits as frequently as patients without progression and an excellent functional status. This is a common bias which often influences the analysis of OS in retrospective oncological studies.

The mechanism that explains the role of MPV/PC ratio in the progression of GB is not yet known. Platelets have various functions in physiological pathways such as coagulation and inflammation. Elevated MPV is considered an early indicator of platelet activation. Activated platelets have procoagulant surfaces that amplify the coagulation reaction (34). The inflammatory reactions and higher thrombotic potential are caused by the release of thromboxane A2 and cytokines (TNF-α, IL-1, IL-6) through large and activated platelets (35). GB patients also often underwent corticosteroid therapy. Corticosteroids are known to increase the platelet count, prolong the platelet survival, and reduce the serum CRP levels (36, 37). This pathophysiological mechanism might also decrease the MPV/PC ratio in GB patients. However, the statistical analysis revealed no significant influence of corticosteroid intake on the MPV/PC ratio in the present series. Furthermore, we have also not observed a significant influence of corticosteroid intake on the baseline levels of serum c-reactive protein. However, it has to be reminded that serum CRP levels might be influenced by several other variables such as cancer, connective tissue disorders, nutritional status, and drugs (38, 39).

In a glioma model, release of soluble CD40 from platelets has been reported; this is a known inhibitor of regulatory T cell recruitment, which promotes an immunosuppressive microenvironment and ultimately a pro-tumor milieu (40). In GB patients, increased CD40 expression on platelets has been described (41). In our subgroup of patients with high-MPV/PC ratio, elevated MPV and decreased platelet counts were found. One potential explanation for the elevated MPV/PC ratio in those patients might be that platelets can be activated in several ways, such as by endotoxins, tumor necrosis, and cancerous cells, which can enhance the consumption of platelets and decrease their numbers. The activation of platelets might damage the endothelium, which would enable cancer cells to invade, and therefore decrease progression-free survival. Additionally, the shorter PFS of patients with an increased MPV/PC ratio could be explained by the release of platelet-derived growth factor and vascular endothelial growth factor through activated platelets during blood clotting, which may induce angiogenesis in those GB patients (42). Furthermore, an *in vivo* and *in vitro* study investigated the platelet aggregation and clotting time in the presence of IDH-1 mutation in gliomas. This study found that mutant IDH-1 gliomas have a decreased platelet activity and increased antithrombotic activity with less common microthrombi within IDH-1 mutant gliomas. Therefore, this pathophysiological mechanism might also explain the superior PFS in those patients with a low MPV/PC ratio and decreased platelet activity (43). In the present study, uni- and multivariate analyses revealed no significant relationship between IDH-1 mutational status and MPV/PC ratio. However, our investigation with regard to the correlation between MPV/PC ratio and IDH-1 mutation status is limited by the low number of patients with an IDH-1 mutant GB (*n* = 16). High relative cerebral blood volumes (rCBV) are associated with angiogenesis and increased mitotic activity in glioblastoma (44). Our retrospective study is limited by rCBV determination not being routinely performed. Future investigations will have to analyze whether increased expression of vascular endothelial growth factor-A (VEGFA) and hyperperfusion according to the rCBV ratio are correlated with an increased MPV/PC ratio. Of primary GBs, 80% were found to express VEGFA. A potential relationship could reveal a subgroup of GB patients who might benefit from a therapy with angiogenesis inhibitors (45). MPV/PC ratio seems to be a promising biomarker for the activity of platelets. Activated peripheral platelets in GB patients can secrete significant relative amounts of sphingosine-1-phosphate compared to healthy controls (41). Sphingosine-1-phosphate directs immune cell migration and fosters monocyte migration from the peripheral blood into the brain. Additionally, sphingosine-1-phosphate is an important part of the transformation of intratumoral macrophages into immunosuppressive tumor-associated macrophages *via* the sphingosine-1-phosphate receptor-1 (46). The pharmacological modulation of sphingosine-1-phosphate levels or its receptor might be a future therapeutic concept in GB therapy. In vitro glioblastoma experiments showed that the sphingosine-1-phosphate analogue fingolimod has anti-proliferative effects on GB cells (47). MPV/PC ratio could be a potential biomarker for these promising therapeutic approaches in order to identify patients with activated platelets.

## Limitations

The present study has several limitations. Data were acquired retrospectively in a single-center study and comparable laboratory data of health subjects for a case-control study were not available in this design. The number of patients included in the high-MPV/PC ratio group was low because the analysis was based on a short study period. Additionally, the high-MPV/PC ratio group included more patients aged 65 years and older. However, statistical analysis revealed a homogeneous distribution regarding demographics, comorbidities and histopathology. Further, this time period (2013-2018) was chosen to compensate for the lack of data with respect of prognostic markers such as IDH mutations and MGMT promoter status in one of the two MPV/PC ratio groups.

## Conclusion

In conclusion, MPV/PC ratio seems to be a potential diagnostic biomarker that independently predicts progression-free survival in glioblastoma patients. This finding might inform the design of future clinical trials investigating immunotherapies downregulating the pro-inflammatory role of platelets in glioblastoma.

## Data Availability Statement

The original contributions presented in the study are included in the article/supplementary material. Further inquiries can be directed to the corresponding author.

## Ethics Statement

The studies involving human participants were reviewed and approved by Ehtics Committee of the University Hospital Bonn. Written informed consent for participation was not required for this study in accordance with the national legislation and the institutional requirements.

## Author Contributions

Data acquisition was performed by SA, MS, and JWa. UH, JWa, and EG performed the data interpretation. Writing and creation of figures were performed by JWa, UH, and EG. Proof reading was done by JWe, PS, UH, EG, and HV. All authors contributed to the article and approved the submitted version.

## Conflict of Interest

The authors declare that the research was conducted in the absence of any commercial or financial relationships that could be construed as a potential conflict of interest.
